# Reference Methods for Measuring Skeletal Muscle Mass: A Critical Perspective

**DOI:** 10.1002/jcsm.70184

**Published:** 2026-01-19

**Authors:** Steven B. Heymsfield, Houchun H. Hu, Edvin Johanssen, Sophia Ramirez, Gabriela de Oliveira Lemos, Maria Cristina Gonzalez, Carla M. Prado, Jonathan P. Bennett

**Affiliations:** ^1^ Pennington Biomedical Research Center LSU System Baton Rouge Louisiana USA; ^2^ Department of Radiology Mayo Clinic Jacksonville Florida USA; ^3^ Antaros Medical Mölndal Sweden; ^4^ Department of Gastroenterology University of São Paulo School of Medicine‐FMUSP Sao Paolo Brazil; ^5^ Postgraduate Program in Nutrition and Food Federal University of Pelotas Pelotas Rio Grande do Sul Brazil; ^6^ Department of Agricultural, Food and Nutritional Science, Faculty of Agricultural, Life and Environmental Sciences University of Alberta Edmonton Alberta Canada; ^7^ Department of Epidemiology University of Hawaiʻi Cancer Center Honolulu Hawaii USA

**Keywords:** body composition, cachexia, frailty, malnutrition, nutritional assessment, obesity, sarcopenia

## Abstract

Skeletal muscle (SM) is an integral organ component in the pathophysiology of many acute and chronic diseases. But is there a ‘gold’ standard or accepted reference method for quantifying the amount and composition of human SM mass? Exploring that question led us to recognize the existence of a SM measurement paradigm that divides methods into two broad categories, in vitro and in vivo. In vitro methods quantify SM mass, weighing intact muscles as part of whole cadaver evaluations, only 51 of which are reported in medical literature with no recent additions. In vivo methods are used to evaluate SM in vivo, and two tiers were revealed in our analyses. An upper tier that included three methods considered ‘reference’ approaches for their accuracy and precision: computed tomography, magnetic resonance imaging and dual‐energy X‐ray absorptiometry. A lower in vivo method tier included bioimpedance analysis, three‐dimensional imaging, several approaches involving creatine metabolism, ultrasound and anthropometry. A feature common to all of the lower tier methods is their need for calibration or validation against reference approaches in the upper in vivo method tier. A critical review of the three in vivo reference methods in the upper tier revealed widely variable SM volume/mass acquisition protocols, image analysis methods and applied terminology. Some reports espouse an upper tier reference method as the ‘gold’ standard while providing minimal details of exactly how and what was measured, thus making replication in follow‐up studies difficult. Any technical issues related to an in vivo reference method are propagated to the in vivo methods in the lower tier that are calibrated or validated against them. Our review of in vivo reference methods of quantifying SM mass and composition led us to two broad recommendations. First, published reports including these reference methods should provide enough details related to acquisition and analysis protocols so that readers can replicate their findings. Second, an effort should be made to apply precise terminology in published reports in order to avoid confusion on exactly what was measured; suggestions are made on definitions of commonly used terms when referring to body composition compartments. Lastly, because there is no consensus on what constitutes a ‘gold’ standard for SM measurement, we suggest expert groups convene in the future to recommend optimum approaches and working guidelines for quantifying muscle mass and composition in vivo.

**Abbreviations**
ALM: appendicular lean mass
ALST: appendicular lean soft tissue
ALSTI: appendicular lean soft tissue index
ASM: appendicular skeletal muscle
AT: adipose tissue
ATFM: adipose tissue free mass
BMC: bone mineral content
BMI: body mass index
BW: weight
Cho: carbohydrate
CSE: chemical shift encoded
CT: computed tomography
DXA: dual‐energy X‐ray absorptiometry
EMCL: extramyocellular lipid
FF: fat fraction
FFM: fat‐free mass
FM: fat mass
H: height
IMAT: intermuscular adipose tissue
IMCL: intramyocellular lipid
intraMAT: intramuscular adipose tissue
LM: lean mass
LST: lean soft tissue
MDC: minimal detectable change
MRI: magnetic resonance imaging
Pro: protein
SAT: subcutaneous adipose tissue
SD: standard deviation
SE: standard error
SM: skeletal muscle

## 1 Introduction

Although the largest body component in most adults [1, 2], skeletal muscle (SM) has only recently attracted substantial research and clinical attention because of its involvement in diseases and conditions such as obesity [3], sarcopenia [4, 5], sarcopenic obesity [6], frailty [7], cachexia [8] and malnutrition [9, 10], all of which are increasing in prevalence. Intense interest among investigators is focusing on how to quantify SM mass and composition in a range of environments from dedicated research facilities to clinical centres and even in field settings.

**FIGURE 1:**
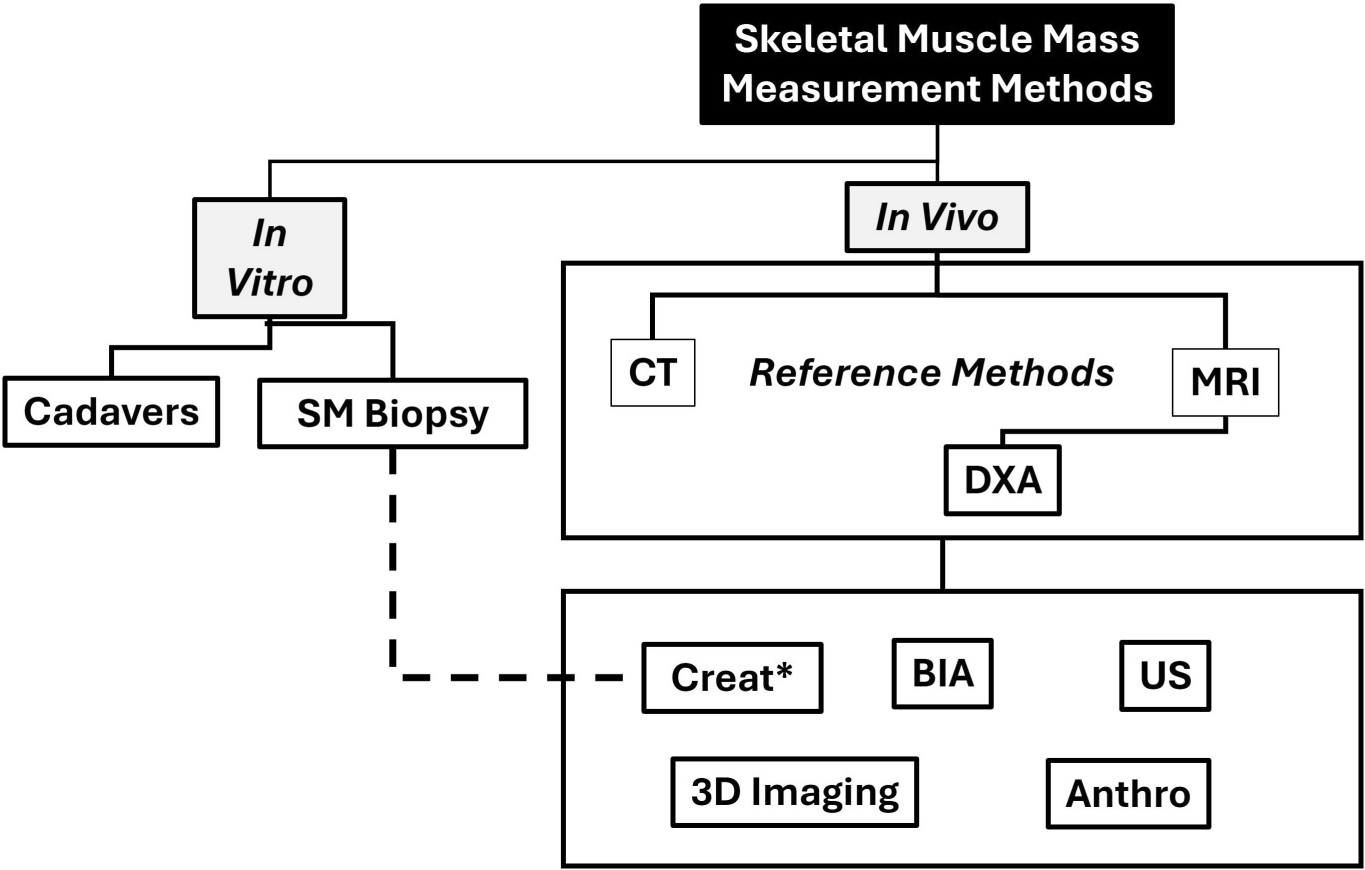
Paradigm outlining methods available for quantifying whole‐body and regional SM volume and mass. In vivo measurement methods are divided into two tiers, an upper tier that includes reference methods against which methods in the lower tier are calibrated or validated against. *The creatine dilution method and model are based on a biopsy sample analysis of creatine concentration [11] and was validated against MRI [12]. ^‡^DXA is often used as a reference method in the form of ALM, ALST and related indices; also, empirical SM prediction equations linking DXA to SM afford the opportunity to use DXA as an SM surrogate reference method [13, 14]. Abbreviations: Anthro, anthropometry; BIA, bioimpedance analysis; Creat, creatine; CT, computed tomography; DXA, dual‐energy X‐ray absorptiometry; MRI, magnetic resonance imaging; SM, skeletal muscle; US, ultrasound.

Multiple methods are available for quantifying SM mass, all of which can be organized for convenience into a simple measurement paradigm as shown in Figure 1. Two broad categories of methods can be defined, ‘in vitro’ and ‘in vivo’ [15, 16]. In vitro methods of evaluating SM mass are conducted ex vivo and involve analysis of cadavers or muscle tissue extracted from living humans. In vivo methods for estimating SM mass include an array of approaches including computed tomography (CT), magnetic resonance imaging (MRI), dual‐energy X‐ray absorptiometry (DXA), bioimpedance analysis (BIA), three‐dimensional (3D) imaging, several approaches involving creatine metabolism, ultrasound (US) and anthropometry [11, 17, 18]. Organization of the SM measurement paradigm as shown in Figure 1 reveals a critical feature of the available in vivo methods: Two tiers are evident, an upper tier that includes three imaging methods that serve as the reference against which the other five methods in the lower tier are calibrated or validated. For example, some BIA systems provide an output of whole‐body and regional SM mass. To generate that information, the manufacturer must reference measured tissue electrical properties such as resistance and reactance against SM components quantified using CT, MRI or DXA in a well‐defined participant sample. The reference sample is then used to develop the system's prediction equations. Thus, the ‘accuracy’ or validity of methods listed in the lower tier all critically rely on the accuracy of the three imaging methods listed in the upper tier. This observation brings into focus several related questions: What exactly do these in vivo reference methods in the upper tier measure with respect to SM volume or closely related mass? Despite differences in these measurement approaches, each with specific technical limitations, do they all give the same estimates of whole‐body and regional SM mass? Is there specific SM‐related terminology that applies to each method? These questions were prompted by the wide array of CT, MRI and DXA reference methods and the frequent lack of detail given in published studies citing them as or implying they are the ‘gold standard’. DXA, reviewed in a later section, is positioned below CT and MRI in the upper in vivo method tier, as it is often used clinically and is recognized as a reference method for estimating SM [19, 20] even though available systems do not measure ‘SM’ as do CT and MRI. Specifically, DXA‐measured appendicular lean mass (ALM) and appendicular lean soft tissue mass (ALST) are often used as proxies for SM mass in clinical settings or as reference estimates for calibrating other in vivo methods shown in the lower tier. Additionally, DXA SM mass prediction equations were developed with ALM and ALST as covariates in equations in which total body MRI measurements served as the reference [13, 14].

Our review is divided into four parts: overview of relevant SM structure and composition along with key definitions; overview of in vitro reference methods; critical assessment of the three in vivo reference methods for estimating SM volume and mass, CT, MRI and DXA; and lastly, we synthesize these observations with an overall perspective on the current status of in vivo reference methods for quantifying SM volume and mass. Each of the sections on in vivo reference methods includes an overview, followed by a detailed examination of relevant image analysis concepts and techniques that provide a framework for our comments on the need for standardizing protocols and terminology.

## 2 Skeletal Muscle Organ

**FIGURE 2:**
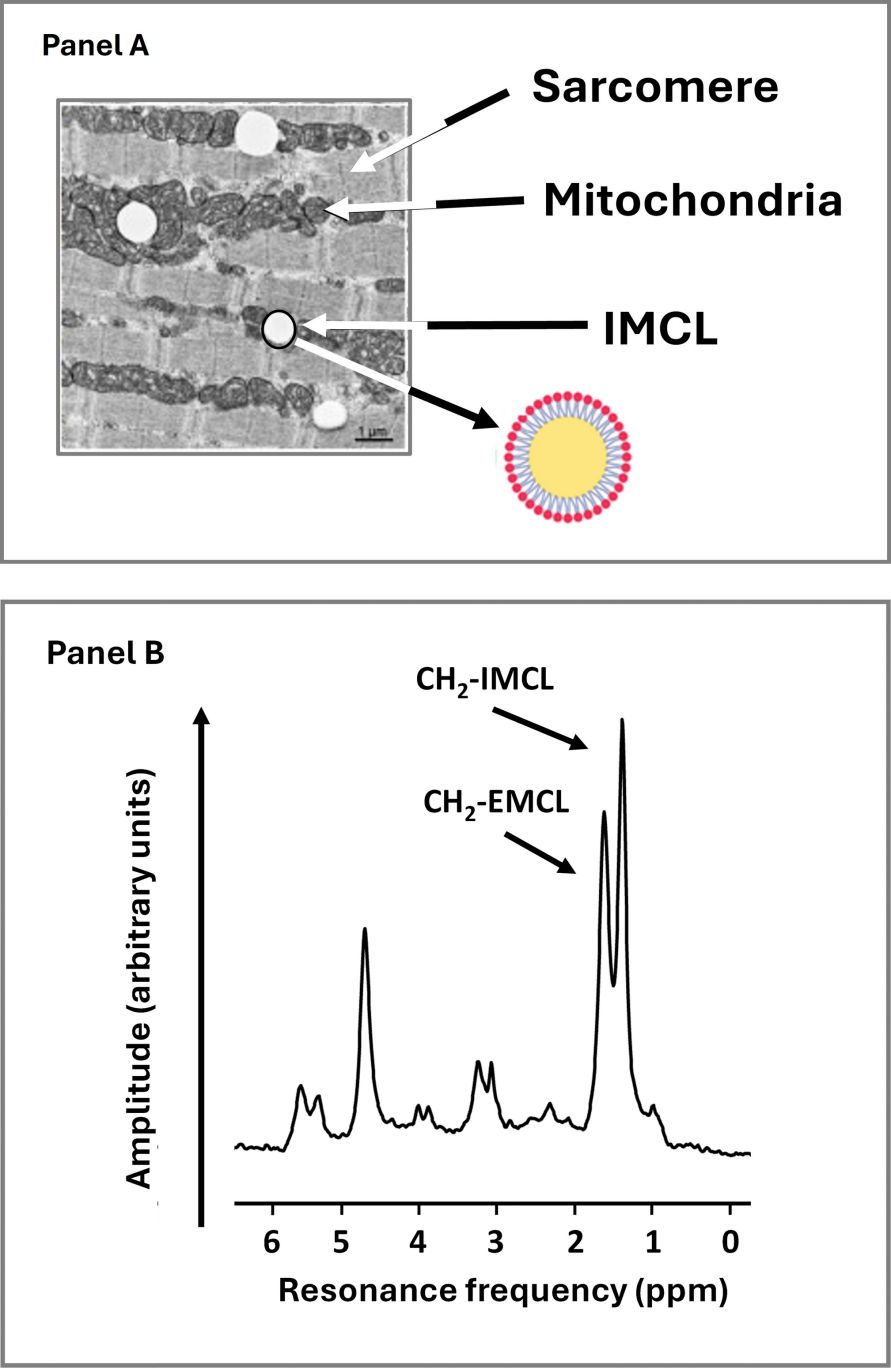
(A) Transmission electron micrograph of a muscle specimen obtained from the medial vastus lateralis that shows white intramyocellular lipid (IMCL) vacuoles adjacent to mitochondria and an illustration of an IMCL droplet with an inner triglyceride (fat) core and outer phospholipid shell. A sarcomere, the smallest functional unit of a myofibril, is shown in the figure adapted from Axelrod et al. [22]. IMCL accounts for about 0.5–2% of wet SM mass in healthy adults [23]. (B) Tibialis anterior muscle ^1^H‐MRS spectrum acquired with a 3T MRI system in a person with Type 2 diabetes. CH_2_‐IMCL, representing intramyocellular lipids stored as droplets within muscle fibres, produces a resonance peak at approximately 1.3 ppm in proton magnetic resonance spectroscopy (^1^H‐MRS). In contrast, CH_2_‐EMCL, representing EMCLs located in adipocytes between muscle fibres, appears at around 1.5 ppm. The small difference in chemical shift is due to the distinct microscopic environments of the lipid protons, particularly their orientation relative to the magnetic field. Courtesy of Vera B. Schrauwen‐Hinderling.

**FIGURE 3:**
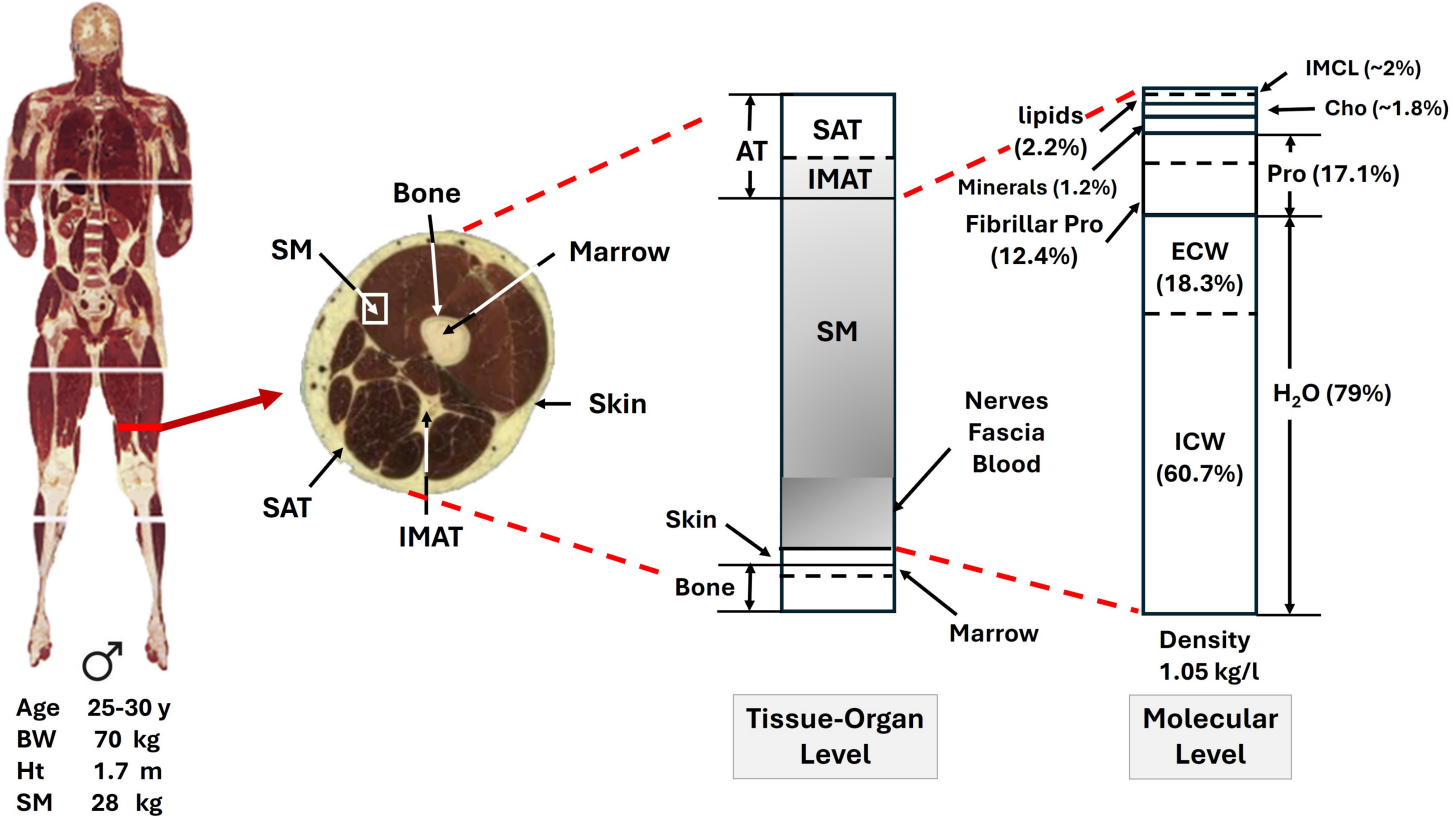
Tissue‐organ and corresponding molecular levels related to SM. The male Visible Human [31] is shown on the left with main 1975 and 1989 Reference Man [1, 32] body characteristics. The tissue‐organ level model describes the main components that are present to the left in the midarm cross‐section. The shaded area represents intact SM as might be observed during cadaver evaluations. The molecular level model on the right reflects the components present in adipose tissue‐free SM. The proportions of molecular‐level components are derived from Reference Man [1]. Carbohydrate and IMCL are labile, and amounts vary with fasting and feeding. Abbreviations: AT, adipose tissue; Cho, carbohydrate; ECW, extracellular water; ICW, intracellular water; IMAT, intermuscular adipose tissue; IMCL, intramyocellular lipid; Pro, protein; SAT, subcutaneous adipose tissue; SM, skeletal muscle.

Skeletal muscle is one of three types of muscle tissue, the other two being smooth and cardiac muscle [21]. The adult human body has over 600 recognized SMs with about 25%–30%, 18%–20% and 55% of total SM mass distributed in the trunk, upper extremities and lower extremities, respectively [1]. Muscle fibres, filled with myofibrils, are bundled into fascicles surrounded by a connective tissue framework. Muscle fibres also include intracellular lipid droplets, referred to as intramyocellular lipids (IMCL), that have an outer phospholipid bilayer and inner core filled with triglycerides that serve as an energy reservoir during periods of increased energy demand (Figure 2A) [24, 25]. The IMCLs, about 0.5–2% of SM mass in healthy adults, can also be quantified with ^1^H‐magnetic resonance spectroscopy (MRS) as shown in Figure 2B. Intact SMs include tendons attached to marrow‐filled bones; nerves; blood vessels; blood; immune cells, myogenic precursors and intermuscular adipose tissue (IMAT); adipocyte collections; and individual adipocytes present between and around SM groups [26, 27]. Some authors separate IMAT into two different types: ‘IMAT’ the adipose tissue beneath fascia and between muscle groups (perimuscular adipose tissue) and ‘intramuscular’ or interstitial adipose tissue, small groups of and individual adipocytes present within muscle fascicles [28, 29]. Intramuscular adipose tissue accounts for the extramyocellular lipid (EMCL) detected with MRS as shown in Figure 2B. We use the term IMAT in the following sections to broadly describe both types of adipose tissue/cells unless otherwise noted specifically as intramuscular adipose tissue. Two types of lipids are thus present in SMs: ‘fat’ or nonpolar lipids that are in the form of triglycerides found in IMAT and IMCL, and polar lipids primarily in the form of sphingolipids and phospholipids that are components of cell membranes [30]. Adipose‐tissue free SM (ATFSM) includes about 12.4% contractile fibrillar proteins and 79% water distributed in the extracellular (18.3%) and intracellular spaces (60.7%) (Figure 3) [1]. Intact SM, in the absence of pathology, is usually assumed to have a density of 1.05 kg/L and includes about 3 mL of blood per kg [1, 32]. Individual and groups of muscles vary widely in composition. Excessive fat accumulation in SM, myosteatosis, reflects relative expansion of IMCL and IMAT triglycerides [29].

## 3 In Vitro Reference Methods

### 3.1 Whole Body

The heart, liver, kidneys, spleen, lungs and brain can all be accurately weighed at the time of autopsy. Large databases with reference values for these organs can be constructed by including people who succumb without a history of acute or chronic disease [33, 34]. In vivo reference methods such as MRI are also available for quantifying the volume and mass of these organs, even in utero [35, 36, 37]. By contrast, very few in vitro estimates of whole‐body SM have been acquired over the past century; the number of cadaver analyses for whole‐body intact SM as part of full cadaver analyses is limited to 51 reported subjects who succumbed from various medical conditions or traumatic accidents [38]. Whole muscles are removed during dissections and thus represent blood‐free intact SM estimates that include all of the components mentioned earlier. Only one cadaver analysis specifically for whole‐body SM mass, and no other organs and tissues, has been reported in recent decades [39] due to the arduous autopsy process requiring 10 expert prosectors working uninterrupted for 15–18 h. Accordingly, the classic 1975 Reference Man estimate of whole‐body SM is based on an in vivo method, 24‐h urine creatinine excretion [1]. Quantifying whole‐body SM is also feasible in animal models, but dissections are tedious, and completely separating muscles from surrounding adipose tissue, bone and other structures is difficult [40]. Reported SM estimates from animal dissections may represent single muscles or whole‐body muscle devoid of tendons, blood and fat (i.e., fat‐free SM) [40]. As a result of the challenges surrounding dissections, our knowledge of whole‐body and regional SM mass in healthy humans thus only began in the 1970s with the introduction of CT and later in the 1980s with MRI [41]. Here, the distinction between in vitro and in vivo body composition methods is a critical one: Cadaver analyses are usually considered the reference methods for organ and tissue mass; skeletal muscle is an exception that requires us to shift focus to in vivo reference methods for accurately quantifying the amount of this relatively large compartment in living humans.

Another in vitro approach involves analysing SM composition obtained by needle biopsy or by excision during surgery to formulate SM prediction models. For example, Baldwin et al. [42] reported the ‘normal’ creatine concentration of wet SM obtained from excised surgical samples in 5 healthy adults. Baldwin et al.'s observations were subsequently used to formulate the mass‐specific constant (4.3 g creatine/kg SM free of blood, fascia and fat) used by Kreisberg et al. [43] and later by Clark et al. [12] to estimate whole‐body SM in adults from the measured creatine dilution space (i.e., SM [kg] = creatine dilution space [g] / 4.3 [g/kg]). Model‐derived estimates of SM such as these require validation in diverse samples of living humans necessitating reference against one of the in vivo methods (e.g., MRI) shown in the upper tier of Figure 1. The creatine dilution method for estimating whole‐body SM therefore is a hybrid approach as classified in the figure, as it includes a model formulated on an in vitro analysis of SM tissue (dashed line in the figure) and validation of the model using an in vivo method, in this case MRI [12]. Alternatively, the creatine dilution space could be calibrated against an in vivo reference method for skeletal muscle mass, thus providing a more realistic model compared to the limitations of an isolated biopsy sample. This suggestion again highlights the critical importance of in vivo reference methods for quantifying whole‐body SM mass.

## 4 In Vitro Reference Methods

### 4.1 Computed Tomography

#### 4.1.1 Overview

The introduction of CT in the early 1970s provided the first opportunity to quantify whole‐body SM at the tissue‐organ body composition level [16, 41]. Cross‐sectional images of SM could be traced by a trained observer using dedicated software allowing for the separation of adipose tissues from muscle bundles and other components [44]. Using this tracing approach, Mitsiopoulos et al. [45] compared the CT‐acquired cross‐sectional areas of SM in the midarm and leg to those evaluated directly in two embalmed cadavers in 1998. Adipose‐tissue‐free SM area (*X* ± SD, 39.7 ± 22.8 cm^2^) did not differ significantly from cadaver estimates (39.5 ± 23.0 cm^2^, *p* = NS), and similarly good agreement was observed between CT and visible cadaver IMAT.

#### 4.1.2 Image Analysis

**FIGURE 4:**
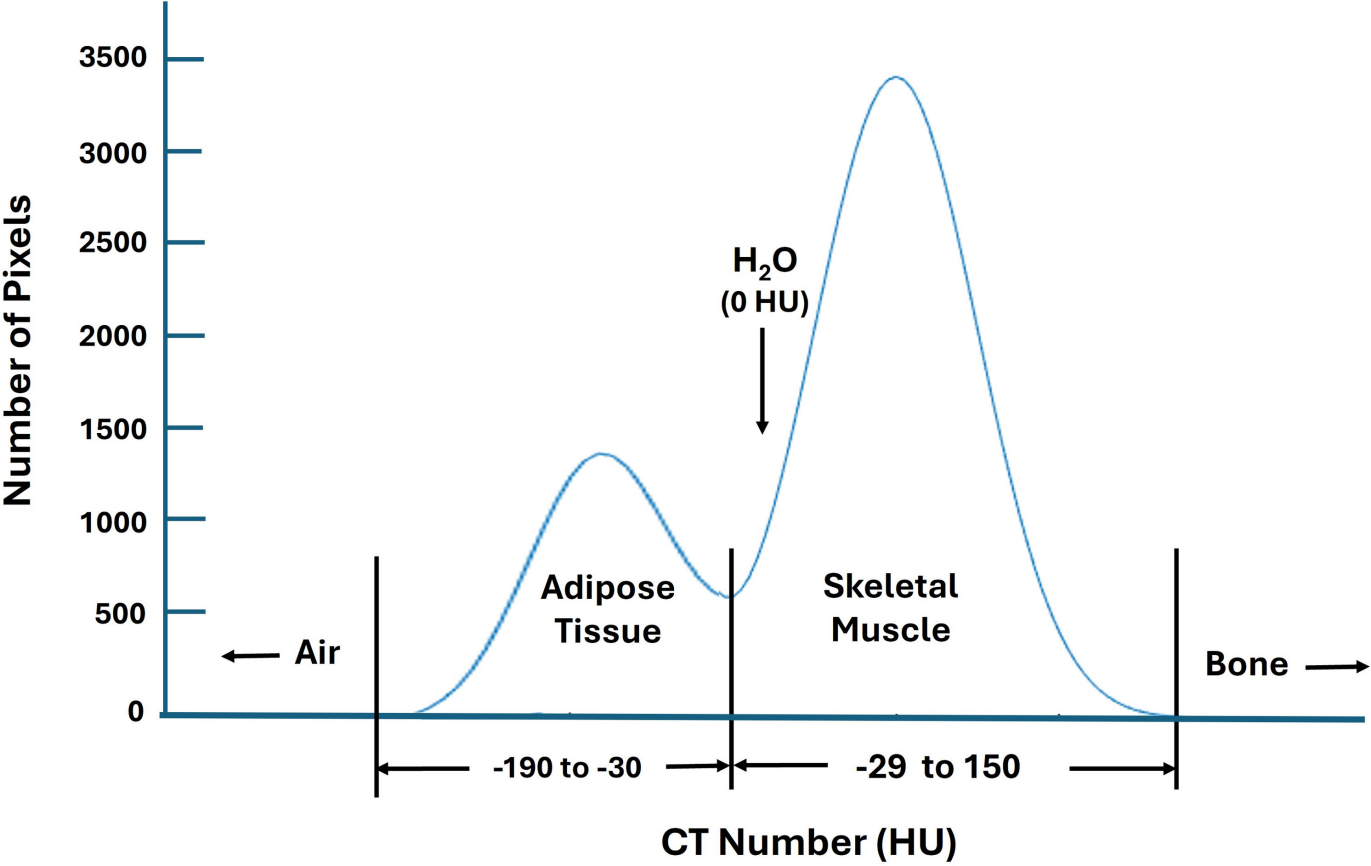
Bimodal distribution of X‐ray attenuation, expressed in Hounsfield units (HU), for a hypothetical SM that includes embedded adipose tissue (i.e., IMAT). Characteristic HU ranges for adipose tissue and SM are shown along with that of water (H_2_O) that is standardized for CT scanners at 0 HU. Air and bone are at the ends of the attenuation range with respective values of −1000 and +1000 HU.

Further refinements in CT technology and analysis protocols were made following the early observations by Mitsiopoulos et al. [45] and others [46]. Among these were advances in thresholding procedures and protocols that allowed the analyst to separate pixels with IMAT from pixels with SM in axial CT slices. Pixels, picture elements, are the two‐dimensional squares that make up the flat CT image. Pixels are separated based on their CT number expressed in Hounsfield units (HU), a numerical value of radiodensity calculated as the linear transformation of evaluated attenuation coefficients [47]. X‐rays passing through tissues are attenuated, the magnitudes of which are determined by several factors, the main ones being physical density and atomic number of interacting molecules [48]. The HU values of air, distilled water and cortical bone in a well‐calibrated system are −1000, 0 and > 1000, respectively. Adipose tissue has a lower HU range (−190 to −30 HU) than water (0 HU), partly due to its smaller physical density (0.92 kg/L vs. 0.997 kg/L at body temperature). Skeletal muscle, which is composed of higher physical density molecules such as protein (17.2% of SM mass; 1.34 kg/L), glycogen (1%–2% of SM mass; 1.63 kg/L) and intracellular fluid (60.7% of SM mass; 1.03 kg/L), has a physical density of 1.05 kg/L and HU range of about −29 to 150 [49]. The scan analyst can outline anatomic SM on the cross‐sectional CT image, remove any bone contributions and plot a histogram of HU values that can then be separated using a slider into SM and adipose tissue pixels according to predefined threshold values (Figure 4). Some evaluated pixels in the figure represent SM towards the right of the bimodal distribution, and others represent solely adipose tissue towards the left of the distribution. There are also intermediate pixels that include both SM and adipocytes, and the resulting attenuation value of these pixels will be proportional to the contributions of each component according to what is referred to as the ‘partial volume’ effect. Moving from right to left in the figure, mean SM radiodensity (HU) decreases with relative increases in adipose tissue. Pixels on the right will include SM along with IMCL and any intramuscular adipocytes that are below the pixel threshold. As the slider moves towards the left with decreasing attenuation values, the pixels increasingly include intramuscular adipocytes along with muscle cells. Moving further to the left additionally captures visible IMAT. Relatively large amounts of intracellular and extracellular fat from IMCL and intramuscular adipocytes, after removal of visible IMAT pixels, are registered as low or negative HU values and are characteristic of the untoward condition referred to as myosteatosis [50]. Using previously proposed SM pixel ranges of −29 to 150 HU and IMAT as pixels ranging from −190 to −30 HU [45], Garcia‐Diez et al. [50] reported a mean SM radiodensity of 50 to 60 HU, and HU values below 50 were considered consistent with myosteatosis of varying magnitudes [50]. Some authors report a normal SM radiodensity of about 48 HU. Thresholding ranges can vary between studies, and thus, there is no standard that defines ‘normal’ or ‘healthy’ SM or SM with minimal amounts of adipose tissue, thus approaching ATFSM.

The radiodensity of SM isolated using thresholding procedures is a measure of muscle fat infiltration as derived from intramuscular adipose tissue and IMCL. CT cannot separate the individual contributions of intramuscular adipose tissue and IMCL due to partial volume effects [25]; a large proportion intramuscular adipose tissue's mass is fat that is detected in the form of EMCLs with MRS. Another approach, reported by Faron et al. [51], is to separate an identified muscle into a ‘fatty’ fraction and remaining tissue. The fatty fraction is defined by the authors as the area within the selected muscle defined by the HU range of −29 to 29 divided by the muscle's total area spanning the HU range of −29 to 100; normal muscle according to this approach was considered the pixel area present between 30 and 100 HU. Edema, present in soft tissues including SM and adipose tissue, can impact the measured HU and is discussed further in Bhadra et al. [52] and Liu et al. [53]. Depending on type, edema has a composition similar to interstitial fluid with an attenuation range of about 0–30 HU.

Depending on protocol, collection of contiguous axial CT images or images with prespecified gaps from head‐to‐toe provide an estimate of total SM volume that can then be converted to mass by assuming a constant muscle physical density of 1.05 kg/L [1] in the absence of tissue pathology. Partial body CT scans are often evaluated opportunistically following imaging studies conducted for clinical purposes [54, 55]. Regional CT SM evaluations are also widely used in clinical studies. For example, iliopsoas muscle area scaled by height squared, the psoas muscle mass index, is a commonly applied CT SM measure [56]. Contemporary refinements in CT imaging include scanning protocols such as spiral CT and automated image analysis software [57, 58].

#### 4.1.3 Need for Standardization

Computed tomography has brought us a long way from the 1975 Reference Man with 28‐kg SM based on 24‐h urine creatinine excretion levels in a small sample of adults [1]. Even if radiation exposure in healthy adults were not a limiting factor in deriving whole‐body estimates of SM, how would we develop updated reference values for whole‐body SM? These questions arise, as CT scan acquisition protocols, muscle groups evaluated and thresholding procedures lack consistency and standardization across studies and in clinical practice [50, 59]. Examples of the details that would be useful in interpreting and replicating studies include scanner manufacturer, tube voltage (kV), exposure (mAs), slice thickness and between‐slice gaps, reconstruction kernel (a process for adjusting image noise and spatial resolution), thresholding procedures and image analysis software, and if manual scan analysis, training of analyst, assumptions made in converting volumes to mass, scanned intervals (e.g., neck‐to‐knee; head, hands and feet included?) and participant positioning, and if longitudinal protocol, positioning of participants between scans. The minimal detectable change (MDC) is the smallest change a measurement method can precisely detect in longitudinal studies. With CT the MDC, influenced by multiple factors, is often not well documented and should be reported in publications. The importance of reporting image acquisition details was emphasized by Troschel et al. [60] who found variation in segmented adipose tissue areas in clinical CT scans as a function of intravenous contrast use, X‐ray tube current‐exposure time product, tube potential and slice thickness. Similarly, Lortie et al. [61] found in a literature review of CT studies that intravenous contrast use and kV variations had marked effects on SM analyses. Estimates of SM radiodensity are thus ideally made with images unenhanced by contrast agents. Amini et al. [62] found in a systematic review of muscle mass and myosteatosis that a sizable percentage of examined CT studies failed to report intravenous contrast use (94%) and slice thickness (64%); diagnostic cut‐points varied widely. Another concern is the lack of standardization of terminology related to SM observations made by CT, and we suggest the use of consistent anatomic taxonomy as shown in Table 1.

**TABLE 1:** Imaging method terminology and suggested protocol reporting.

Term	Abbreviation	Definition
Terminology		
Adipose tissue	AT	The intact tissue that in healthy adults includes about 80%–90% of its mass as triglyceride (fat).
Adipose tissue free mass	ATFM	Derived as body weight minus whole‐body AT.
Adipose tissue free skeletal muscle mass	ATFSM	Derived as total SM minus IMAT.
Appendicular lean soft tissue mass	ALST	The DXA‐measured LST mass present in appendages.
Appendicular lean soft tissue index	ALSTI	The ratio derived by dividing ALST by height^2^.
Appendicular lean mass	ALM	The difference between DXA‐measured appendicular total mass and fat mass; alternatively, the sum of appendicular ALST and BMC.
Appendicular lean mass index	ALMI	The ratio derived by dividing ALM by height^2^.
Appendicular skeletal muscle	ASM	The SM present in the extremities.
Bone mineral content	BMC	The DXA‐measured mineral portion of bone.
Extramyocellular lipid	EMCL	Lipid in the form of triglycerides present in adipocytes within muscle fascicles.
Fat fraction	FF	Fat fraction in MRI is derived as the fat proton signals divided by the sum of the fat and water proton signals.
Fat‐free mass	FFM	Derived as body weight minus FM.
Fat‐free skeletal muscle mass	FFSM	Derived as total SM minus total muscle fat (IMAT & IMCL).
Fat mass	FM	Neutral lipids, mainly in the form of triglycerides, such as those stored in adipose tissue used as a source of metabolic energy.
Intermuscular adipose tissue	IMAT	Single or multiple grouped adipocytes found within skeletal muscles [26]. Some reports describe intramuscular adipose tissue, part of IMAT, as adipocytes present within muscle fascicles. A consensus is lacking on this terminology.
Intramyocellular lipid	IMCL	The intramyocellular lipid, mainly neutral triglyceride (fat), within micelles enclosed by a phospholipid bilayer present in skeletal muscle fibres.
Lean mass	LM	Equivalent to FFM: The difference between total body mass and fat mass.
Lean soft tissue mass	LST	DXA‐measured mass excluding fat and bone mineral.
PDFF	Proton Density Fat Fraction	Serves as a standardized MRI‐based biomarker of tissue fat concentration.
Skeletal muscle	SM	The intact organ‐tissue level component that includes over 600 individual muscles.

*Note:* Adapted from [63, 64, 65].

Abbreviations: DXA, dual‐energy X‐ray absorptiometry; MRI, magnetic resonance imaging.

### 4.2 Magnetic Resonance Imaging

#### 4.2.1 Overview

Magnetic resonance imaging was introduced to the clinical setting in the decade following CT [41]. There are two MRI approaches that are commonly used to evaluate SM: T1‐weighted (T1w) imaging and chemical‐shift‐encoded (CSE) water‐fat MRI [66, 67]. T1w and CSE‐MRI methods traditionally have been used for body composition profiling, particularly focusing on adipose tissue. Here, we shift the emphasis specifically to SM while continuing to review adipose tissue as an integral feature of intact muscles.

#### 4.2.2 Image Analysis

T1w images acquired in the transverse (axial) orientation are commonly employed to measure SM using 2D slices with interslice gaps or 3D contiguous volumes. Adipose tissue, comprised mainly of triglycerides, has a proton T1 relaxation time in T1w MRI that is significantly shorter than the water protons present in lean tissues. Adipose tissue is therefore hyperintense (brighter) in a T1w image relative to SM and other organs. These T1w signal contrast differences in pixel intensity facilitate segmentation of various tissue and organ components. The signal intensity histograms generated from T1w images are analogous to the CT HU histograms generated by the analysis of intact SM (Figure 4), including IMAT. The analyst or automated software can separate out the SM components present in a T1w histogram according to the varying signal intensities of adipose tissue and SM similar to CT as described earlier. With T1w MRI, histogram thresholding of signal intensity is effectively defined by discrete cutoff values. Pixels with signal intensities greater than the cutoff value are treated as adipose tissue, whereas signal intensities from pixels less than the cutoff value and up to a specified range are considered SM. The number of SM pixels counted is then multiplied by pixel area to produce an estimate of SM area; alternatively, counted pixels x voxel volume ➔ SM volume, with voxels defined as volume elements. Mitsiopoulos et al. [45] found close agreement between T1w MRI‐measured adipose‐tissue free SM cross‐sectional areas (38.9 ± 22.3 cm^2^) and those evaluated in embalmed cadaver limbs (39.5 ± 23.0 cm^2^, *p* = NS); good agreement was also observed between MRI and cadaver IMAT estimates.

CSE‐MRI, historically referred to as Dixon water‐fat separation MRI, has been adopted in many body composition protocols over the past decade [67]. Unlike T1w MRI, the signal within each acquired voxel is explicitly separated into water and triglyceride (fat) components in CSE‐MRI. In its most basic form, this separation can be achieved using a so‐called two‐point Dixon technique [68] that relies on a pair of acquired images where the water and fat signals are in phase and out of phase; the phase relationship arises from the resonance frequency difference between water and fat protons. More advanced six‐point Dixon techniques [69] acquire images at six echo times to model and correct confounding factors such as the multiple resonance peaks of fat, signal decay and field inhomogeneities. Although six‐point Dixon methods yield more accurate and robust water‐fat separation and are preferred for quantification, they come at the cost of longer acquisition times and increased computational complexity. From separated water and fat signals, fat fraction (FF = [fat signal] / [fat + water signals]) maps can be generated. An alternative implementation is to generate quantitative FF maps derived exclusively from the isolated fat signal using signal intensities within subcutaneous adipose tissue as an internal reference standard such that FF = [fat signal] / [fat in subcutaneous adipose tissue signal] [70, 71].

As with the variation in technical aspects of CT, methods for deriving FF differ according to manufacturer, scanner, imaging sequence and reconstruction software [72]. Reeder et al. [69] summarized the biological, physical and technical factors influencing magnetic resonance fat and water signals that can confound the accuracy of FF as a measure of tissue triglyceride; an alternative, proton density fat fraction, was recommended as an unbiased alternative. Regardless of origin, FF is expressed as a percentage ranging from 0% to 100%, with subcutaneous and visceral depots of white adipose tissue typically exhibiting high FF values, often above 80% [71], thus allowing fat‐containing adipose tissues to be readily distinguished from lean tissues. Fat fraction, in this context, thus serves as a thresholding parameter analogous to the HU scale in CT imaging or intensity thresholds in T1w MRI. In contrast to T1w image analysis where the pixel signal intensity and histograms have arbitrary slider scales depending on the system and pulse sequences used, CSE‐MRI with the associated FF is more reproducible as the scale is normalized as 0%–100% [73].

The thresholding approach is sometimes employed for identifying fatty infiltration within SM [50, 74], based on separating voxels with a FF of < 50% reflecting SM, from those with a FF > 50% reflecting IMAT [75, 76]. According to this approach, muscle fat infiltration is defined as the mean FF of the SM region [69, 72, 76]. However, this estimate will not include intramuscular adipose tissue from regions where it is most concentrated, such as in fat streaks, since such regions may be above the 50% threshold. An alternative is to base segmentation on anatomical muscle features [77]. Anatomically guided approaches were previously far more labour‐intensive than threshold‐based methods, but with the emergence of deep learning–based Artificial Intelligence techniques such assessments of SM fat infiltration are now also feasible at scale [78].

Edema and lymphedema, frequent presentations in clinical populations, can be visualized in soft tissues that include SM by varying MRI pulse sequences [79, 80]. These imaging methods are not reviewed in the current report. MRI system field strengths also vary; typically, devices are either 1.5 or 3 Tesla. More recently, low‐field strength magnets have proved useful in body composition evaluations. The obtained images at 0.55 Tesla are of good quality such that automated postprocessing is feasible and results are repeatable [81].

The major advantage of MRI over CT is the lack of radiation exposure, thus allowing acquisition of scans across the whole lifespan and multiple evaluations in longitudinal studies. Single MRI slices at specific anatomic sites can be used as estimates for whole‐body SM [82]. Image analysis methods have evolved from early manual segmentation protocols [44, 61] to semiquantitative and fully automated approaches, the latter now aided by Artificial Intelligence [58, 66, 67, 69, 72]. Measured SM volumes can be converted to mass estimates assuming SM's physical density, as reported in healthy adults, is 1.05 kg/L [1].

#### 4.2.3 Need for Standardization

As with CT, protocol and image analysis details in reports of SM as evaluated with MRI are often lacking. As an example, the pivotal validation study of the D_3_‐creatine dilution method for measuring SM mass included MRI as the reference but provided no details of the scanning protocol or image analysis method [12]. Replication of this study would thus not be possible without communicating directly with the author on details of the MRI protocol. Accordingly, reports of SM measurements with MRI should specify details of the scanning and participant protocols such as manufacturer, device name, magnet strength, sequence, repetition time, echo time, field of view (FOV), bandwidth, slice thickness, slice gap, repetition time, flip angle, MDC, participant positioning and, if images are manually segmented, analyst training.

### 4.3 Dual‐Energy X‐Ray Absorptiometry

#### 4.3.1 Overview

The radioactive isotope ^153^Gd emits photons at two energy levels, one at 44 keV and the other at 100 keV [83]. Gadolinium's emitted photons are differentially absorbed when passing through tissues, and the magnitude of these relative effects is distinct for each element. Mazess et al. [84] exploited this phenomenon in 1970 when they reported that fat, lean soft tissue (LST) and bone each have distinct gadolinium photon absorption signatures due to their differing contents of elements such as carbon, sodium and calcium. Each tissue pixel in a dual‐photon absorptiometry (DPA) scan, as it became known, could be separated into one of two types, soft tissue without bone and soft tissue plus bone. The differential photon signature in pixels without bone allowed the authors to separate contents into fat and LST. Mathematical models could then be used to solve for the proportion of fat and LST present in the soft tissue overlying bone [63, 64, 83]. These observations led to the introduction of DPA as a method for evaluating body composition, dividing total body mass into three components, fat, LST and bone mineral content (BMC) [83]. The DPA method relied on the assumption that fat, LST and BMC each had a stable and constant attenuation signature in relation to the two applied gadolinium photon energies [83]. The dual‐photon gadolinium‐based DPA method was soon supplanted by similar concept DXA systems that generated two effective X‐ray photon peaks [85, 86]. Early DXA systems were calibrated using the saturated triglyceride stearic acid as fat, water enriched with 8.6% electrolytes as LST and bone ash or calcium hydroxy apatite as BMC [83, 85]. Aluminium, polyethylene and other similar solid and transportable materials are now used by manufacturers and in clinical settings to calibrate DXA systems [85].

#### 4.3.2 Image Analysis

**FIGURE 5:**
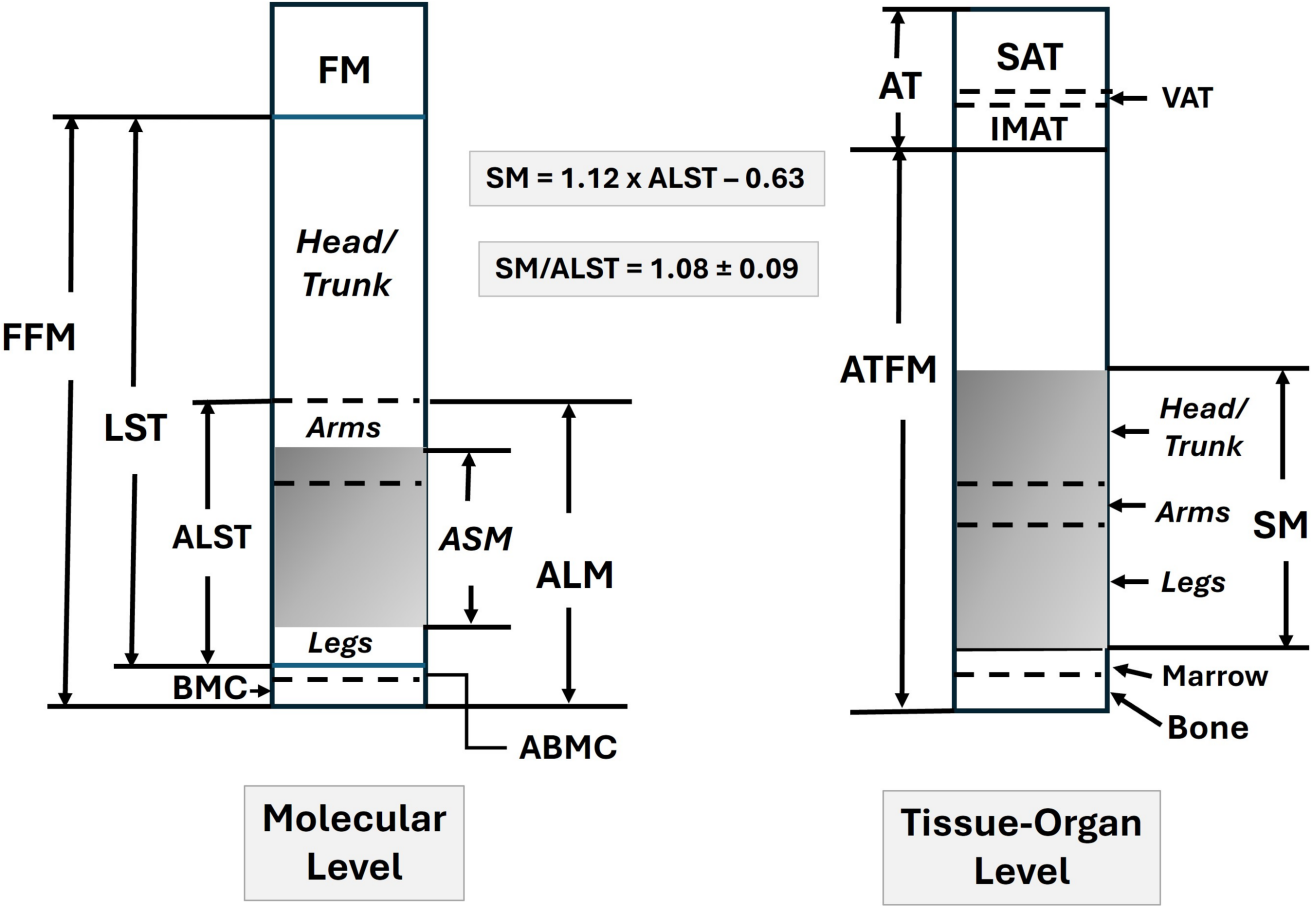
Molecular level DXA whole‐body model and corresponding tissue‐organ level model. Skeletal muscle mass (kg) can be predicted by empirical equations with ALST (kg) as the predictor variable, an example of which is shown in the figure [87]. The ratio of whole‐body SM to ALST, also shown (*X* ± SD) in the figure, is approximately 1.0, and ALST is therefore frequently used as a surrogate for whole‐body SM. The shaded area at the molecular level represents the proportion of ALST as ASM. The shaded area at the tissue‐organ level represents the proportion of ATFM as SM. Abbreviations: ABMC, appendicular bone mineral content; ALM, appendicular lean mass; ALST, appendicular lean soft tissue; ASM, appendicular skeletal muscle; AT, adipose tissue; ATFM, adipose tissue free mass; BMC, bone mineral content; FFM, fat‐free mass; FM, fat mass; IMAT, intermuscular adipose tissue; LST, lean soft tissue; SAT, subcutaneous adipose tissue; SM, skeletal muscle; VAT, visceral adipose tissue.

Modern DXA systems, employing various models and assumptions, can quantify fat, LST and BMC in the whole body and in regions including the head, neck and trunk, arms, and legs. Of the total extremity LST, about 68% is fat‐ and BMC‐free SM [87] and, as noted earlier, ~18%–20% and ~55% of whole‐body SM is distributed in the arms and legs, respectively [1, 63, 64] (Figure 5). These observations give rise to two methods of estimating whole‐body SM. First, because of the large contribution of appendicular SM (ASM) to whole‐body SM, empirical SM prediction models can be developed as shown by the example in the figure [87]. Second, ALST and ALM (ALST + appendicular BMC) are about the same absolute magnitude as whole‐body SM (Figure, SM/ALST, ~1.08) and can be used as SM surrogates alone or adjusted for height [4, 9]. DXA SM estimates, often in the form of ALST or ALM, serve as the reference for calibration or validation of other methods such as BIA [88, 89] and 3D imaging [90].

DXA systems evaluate fat mass and not adipose tissue volume or mass as with some CT and MRI protocols. DXA measurements can be used, however, to predict components such as visceral adipose tissue based on equations using CT or MRI as the reference [91]. Although early DXA systems were calibrated against chemical moieties such as stearic acid [85], a triglyceride, if and to what extent current DXA fat estimates include other lipids (e.g., membrane phospholipids), is uncertain. Extremity lean soft tissue (i.e., ALST) is fat‐ and BMC‐free but includes all of the nonfat contents of SM, epidermis, adipose tissue, bone marrow, blood and the nonmineral portions of bone (Figure 5). The proportions of these constituents that comprise ALST are not constant but can vary with multiple factors including age and level of adiposity. As with visceral adipose tissue, sometimes referred to as visceral fat, it is feasible to predict IMAT from DXA measurements using data acquired from CT or MRI as the reference [92].

#### 4.3.3 Need for Standardization

There are two main manufacturers of DXA systems (Hologic and GE Lunar), and they often update their devices and software that can impact body composition measurements [93]. This variability between devices and software versions needs to be considered when conducting multicentre and longitudinal studies. Publications reporting DXA body composition observations should, accordingly, provide system manufacturer, software version, scan mode, analytical considerations (e.g., use of mirroring or artefact removal), measurement precision and MDC for main outcome measures in longitudinal studies. DXA terms are also often misapplied in the research literature, for example, LST described as ‘lean body mass’ [30, 63]. Critical reporting details for DXA‐derived SM include whether estimates were calculated using a specific prediction equation (with citation and calibration system noted) or if proxy measures such as ALST were used. The suggested standard DXA terminology is presented in Table 1.

## 5 Perspective

Two eras in body composition analysis crossed in the early 1970s: Reference Man [1] was published that based estimates of SM mass on the five‐decade‐old urinary creatinine method [17], and Godfrey Hounsfield introduced computerized axial tomography [41] that provided investigators with the first in vivo reference method for quantifying whole‐body SM volume and mass. Magnetic resonance imaging, DPA and DXA soon followed, additional novel in vivo reference approaches for evaluating SM mass in a wide range of clinical and research settings [41]. A vast literature has evolved on methods of quantifying SM mass with these tools that are central to calibrating and validating other in vivo methods for measuring SM mass. This abundance of methods for quantifying SM mass in vivo has given rise to multiple approaches for defining and quantifying SM mass, leaving uncertainty if and what approach is the ‘gold standard’.

**FIGURE 6:**
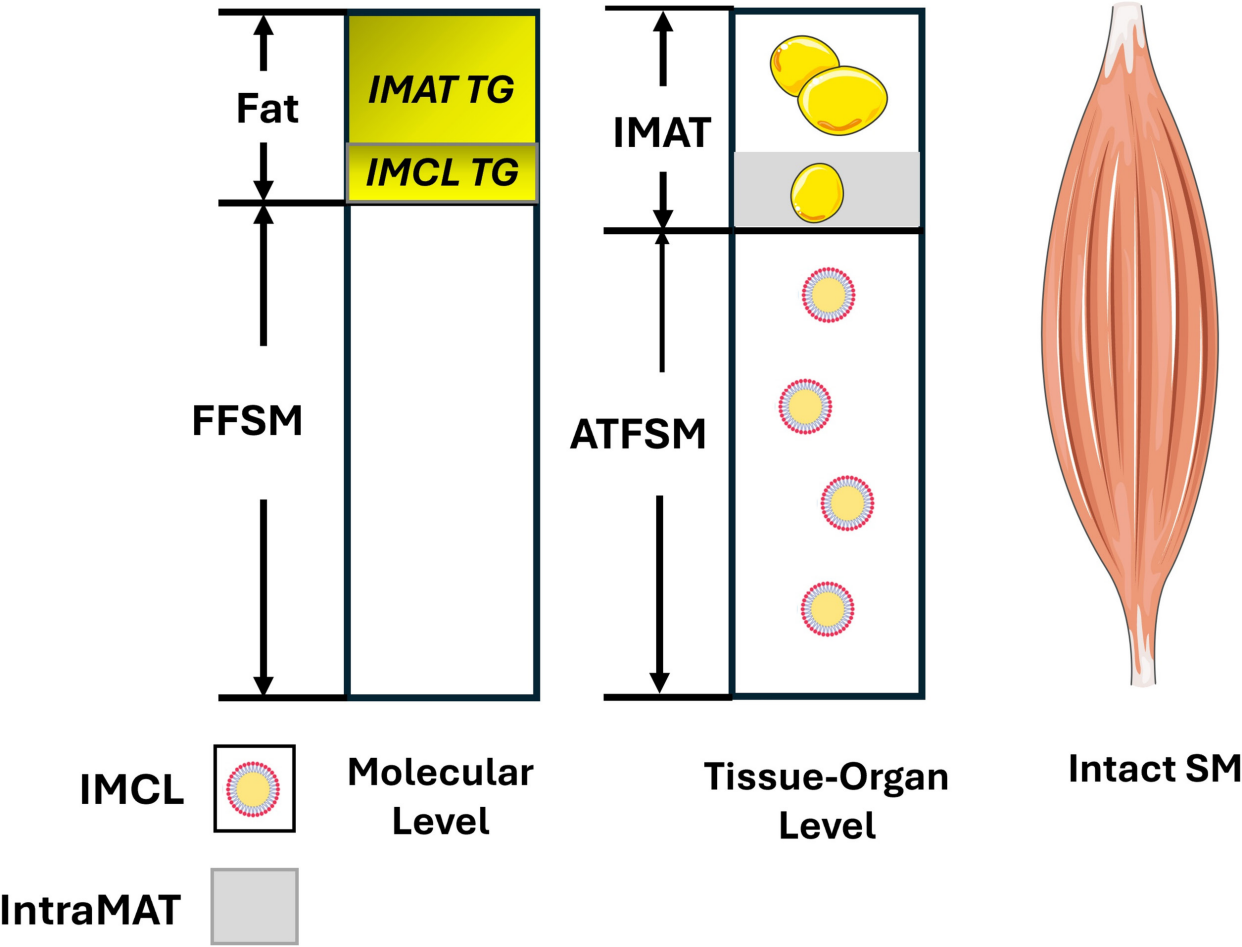
Three hypothetical definitions of a standard reference SM are displayed in the figure: intact SM; SM at the tissue‐organ level; and SM at the molecular body composition level. Grey shading shows the proportion of IMAT as intramuscular adipose tissue (IntraMAT). Yellow shading denotes fat (triglyceride). Abbreviations: ATFSM, adipose tissue‐free skeletal muscle; FFSM fat‐free skeletal muscle; IMAT, intermuscular adipose tissue; IMCL, intramyocellular lipid; SM, skeletal muscle; TG, triglyceride.

Specifically, our review shows that the amount of SM measured with in vivo reference methods is variable depending on multiple factors, and hence, there is no single accepted ‘gold standard’ against which other in vivo methods are calibrated or validated. Recognizing the technical differences between methods available across CT and MRI systems is essential for interpreting SM measurement concordance between studies. Here, we can consider three hypothetical definitions of a reference SM mass as shown in Figure 6. The first is intact SM that includes the components that would be present when a muscle is excised in a cadaver study. Intact muscle volume and mass can be quantified with the CT and MRI methods, and reference values could be developed in well‐defined samples of children and adults. The second hypothetical reference muscle is at the tissue‐organ level and for simplicity is divided into adipose tissue and ATFSM that includes IMCL. This model includes the SM components needed to generate muscle force and sustain metabolic activity and is variously described in the literature as lean or pure muscle tissue. The challenge in implementing this model is dialing out with CT or MRI all adipose tissue and adipocytes while leaving behind ATFSM. The third hypothetical model, at the molecular level, also divides muscle into two components, fat and fat‐free SM. The fat component in this model includes IMAT and IMCL triglycerides and is analogous to the two‐component whole‐body molecular level model that divides body mass into fat and FFM. This third model of reference SM is amenable to analysis with CSE‐MRI, the challenge again to completely dial out fat from remaining SM. DXA estimates fat‐ and BMC‐free ALST, conceptually fat‐free ASM, although measured values now include non‐SM contributions from other constituents. Each of the models has a level of complexity for analysis but suggests theoretical targets to strive for when setting standardized analysis parameters yet to be established. An unknown in this context is if some proportion of SM as IMAT or IMCL and their respective associated fat contents is associated with optimum mechanical and metabolic function.

Setting standardized imaging protocols and terminology has value to the scientific community that is increasingly focusing on SM as a relevant component in multiple increasingly prevalent acute and chronic diseases. To start with, we suggest investigators report details of their imaging protocols and use standard terminology as recommended in our review and those of others [19, 65, 94]. Next, there is a need for expert groups with diverse backgrounds and expertise that have the qualifications to weigh in on standardizing the clinical and technical imaging details involved. This task can be facilitated by organizations or societies that are involved with biomedical imaging and the multiple acute and chronic diseases now recognized to involve SM mass and function. Adopting these measures will facilitate accurate communication among investigators evaluating SM mass and composition and ensure that studies can be critically analysed and replicated.

## Funding

This work was partially supported by the National Institutes of Health NORC Center Grants P30DK072476, Pennington/Louisiana; P30DK040561, Harvard; and R01DK109008. C.M.P. is partially supported by the Canada Research Chairs Program.

## Ethics Statement

The authors have nothing to report.

## Conflicts of Interest

S.B.H. serves on the Medical Advisory Boards of Tanita Corporation, Novo Nordisk, Lilly, Abbott, Regeneron and Medifast. E.J. is an employee of Antaros, a company involved in analysing biomedical images. C.M.P. has previously received honoraria and/or paid consultancy from Abbott Nutrition, Nutricia, Nestle Health Science, AMRA medical and Novo Nordisk. The authors and their close relatives and their professional associates have no financial interests in the study outcome nor do they serve as an officer, director, member, owner, trustee or employee of an organization with a financial interest in the outcome or as an expert witness, advisor, consultant or public advocate on behalf of an organization with a financial interest in the study outcome.

## Data Availability

Data described in the manuscript will be made available upon request pending application and approval by the investigators.
