# Correction to ‘Reference Methods for Measuring Skeletal Muscle Mass: A Critical Perspective’

**DOI:** 10.1002/jcsm.70236

**Published:** 2026-02-11

S. B. Heymsfield , H. H. Hu , E. Johanssen , et al., “Reference Methods for Measuring Skeletal Muscle Mass: A Critical Perspective,” Journal of Cachexia, Sarcopenia and Muscle 17, no. 1 (2026): e70184, 10.1002/jcsm.70184.41555669 PMC12816762

The author's name ‘Edvin Johanssen’ should be revised to ‘Edvin Johansson’.

Additionally, following statement should be included in the Conflicts of Interest section:

‘M.C.G. has received honoraria and/or paid consultancy from Abbott Nutrition, Nutricia, and Nestlé Health Science Brazil’.

These changes have now been updated in the original article.

We apologize for this error.

